# Bioinformatics Application with Kubeflow for Batch Processing in Clouds

**DOI:** 10.1007/978-3-030-59851-8_24

**Published:** 2020-09-15

**Authors:** David Yu Yuan, Tony Wildish

**Affiliations:** 8 University of Tennessee at Knoxville, Knowville, TN USA; 9 Department of Mathematics, KIT für Technologie Karlsruhe, Karlsruhe, Baden-Württemberg Germany; 10 Computational Science, Helmholtz-Zentrum Dresden Rossendorf, Dresden, Sachsen Germany; 11 Extreme Computing Research Center, King Abdullah University of Science and Technology, Thuwal, Saudi Arabia; Technology and Science Integration, European Bioinformatics Institute, European Molecular Biology Laboratory, Hinxton, UK

**Keywords:** Kubernetes, Kubeflow, Workflow, Container orchestration, Deployment, Clouds, Data management, Monitoring, OpenStack, Google Cloud Platform, Amazon Web Services

## Abstract

Bioinformatics pipelines make extensive use of HPC batch processing. The rapid growth of data volumes and computational complexity, especially for modern applications such as machine learning algorithms, imposes significant challenges to local HPC facilities. Many attempts have been made to burst HPC batch processing into clouds with virtual machines. They all suffer from some common issues, for example: very high overhead, slow to scale up and slow to scale down, and nearly impossible to be cloud-agnostic.

We have successfully deployed and run several pipelines on Kubernetes in OpenStack, Google Cloud Platform and Amazon Web Services. In particular, we use Kubeflow on top of Kubernetes for more sophisticated job scheduling, workflow management, and first class support for machine learning. We choose Kubeflow/Kubernetes to avoid the overhead of provisioning of virtual machines, to achieve rapid scaling with containers, and to be truly cloud-agnostic in all cloud environments.

Kubeflow on Kubernetes also creates some new challenges in deployment, data access, performance monitoring, etc. We will discuss the details of these challenges and provide our solutions. We will demonstrate how our solutions work across all three very different clouds for both classical pipelines and new ones for machine learning.

## Introduction

Bioinformatics pipelines make extensive use of HPC batch processing farms. The data size is growing exponentially in Terabyte to Petabyte range. The computational complexity is also growing rapidly with job duration reaching weeks to months. HPC facilities can no longer satisfy these rapidly growing requirements. With the modern applications of machine learning algorithms, GPUs become critical for batch processing, but long wait times for GPU batch queues are common. With rapidly changing GPU models, high unit price and long procurement cycles, it is impossible to run some pipelines simply due to the lack of specific GPU models on premises.

HPC-in-the-cloud solutions provide VM-based workflow management. Open source tools like Cluster-in-the-Cloud are more portable, but also need lots of maintenance. In general, batch clusters are complex to configure for general users, and don’t take good advantage of cloud-native capability. We tried implementations on different clouds: Google Cloud Platform, Microsoft Azure and Oracle Cloud. The solutions are very cloud-specific, and thus unportable.

Container and its orchestration engine Kubernetes is the obvious choice to overcome issues with VM-based batch solutions in clouds. The basic Kubernetes job framework is insufficient for Bioinformatics pipelines. It is more of a framework for frameworks. Kubeflow [1] is a comprehensive and cloud-agnostic workflow engine on Kubernetes. It is designed for machine learning workflows but generic enough to run any workflow on Kubernetes in a simple, portable and scalable fashion.

In this article, we are to deploy Kubernetes and Kubeflow to run two pipelines: one for classic pipeline and the other for machine learning, targeting three clouds: a private cloud based on OpenStack (OSK), and two public clouds of Google Cloud Platform (GCP) and Amazon Web Services (AWS). Although Kubernetes has become the de facto standard on almost all major clouds, there are also some new challenges in data access, performance monitoring, and GPU etc. We will discuss the details of these challenges and our solutions. We will demonstrate how our solutions work across all three very different clouds for both classical pipelines and new ones for machine learning.

## Method

Docker and Kubernetes have become the de facto standard for container and container orchestration. All major cloud providers and operating systems provide first class support for them. In our previous investigation, we have confirmed that Bioinformatics pipelines can be migrated from HPC into public clouds with ease. In addition, the resulting Kubernetes clusters are almost identical in Google, Amazon and Microsoft [2]. Together, Docker and Kubernetes become universal platforms for Infrastructure-as-a-Service (IaaS) for Bioinformatics pipelines and other workloads.

Kubernetes has a job framework built into its APIs [3]. However, it is in its infancy and incapable to support complex pipelines for Bioinformatics. Google, together with many other major cloud vendors, have just started a new workflow engine, Kubeflow, on Kubernetes to make ML simple, portable and scalable. Kubeflow shows promise as a platform to manage the workflows of Bioinformatics pipelines with efficiency, scalability and portability. In this section, we will focus on the challenges, temporary and long term, and our solutions to address them.

### Deployment

We have Kubernetes clusters for HPC on three clouds: OSK, GCP and AWS. We run Rancher Kubernetes Engine (RKE) [4] on OSK. Public clouds have their Kubernetes engines built in: GKE on GCP and EKS on AWS. Kubernetes provides a good solution for computing. It is relatively weak on integration with storage and network.

We then deployed Kubeflow for batch processing. There are two categories of deployment for Kubeflow:

1. Cloud-agnostic: the deployment scripts are maintained by the open source community or third party, for example the first two scripts in the table [5]. They require the Kubernetes cluster created first.
2. Cloud-specific: the deployment scripts are maintained by cloud providers such as GCP, AWS, IBM and OpenShift. Microsoft is using the community maintained script at present.

**Fig. 1.**
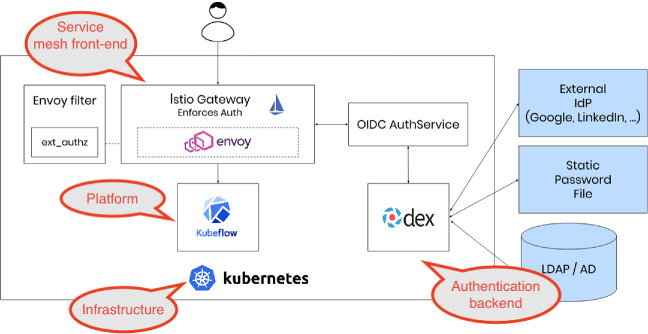
Multi-user, auth-enabled Kubeflow was modified from Kubeflow documentation (https://www.kubeflow.org/docs/started/k8s/kfctl-istio-dex/) under CC BY 4.0 license.

We have been using both cloud-agnostic and cloud-specific scripts. The cloud-agnostic script is completely portable. We are able to deploy Kubeflow on OpenStack, GCP and AWS without modification. This would reduce our operational cost in production and the implementation of the hybrid cloud strategy. The cloud-specific script provides tight integration with the underlying cloud infrastructure. The benefit to end users is minimal at this point. Therefore, we have chosen the cloud-agnostic script (kfctl_istio_dex.v1.0.0.yaml) [6] for all of our three clouds. It provides a consistent mechanism for authentication and authorization [7] as shown in Fig. 1.

**Storage.** Cloud providers only support a very small subset of Volume Plugins, with few overlaps. They all support ReadWriteOnce mode. About 60% of them support ReadOnlyMany. Only 30% of them support the ReadWriteMany model [8]. Bioinformatics pipelines almost always assume local access to POSIX-like file systems for both read and write, so we use an NFS persistent volume as a workaround to make our pipelines cloud-agnostic.

NFS has many limitations in security, performance, scalability and, to a certain extent, data integrity. We only use it to pass a small amount of intermediate data between tasks in the same pipeline. For temporary files within a task, we use the default Storage Classes to create Persistent Volumes. The Volume Plugins for the default Storage Classes always support ReadWriteOnce. The Persistent Volume Claims (PVCs) always use the default Storage Classes if omitted. This makes the PVC manifest syntactically identical in all the clouds to create temporary storage for reading and writing within a task. We mount emptyDir in a pod for caching. If cache is small, we set emptyDir.medium field to “Memory” for fast access as the tmpfs mounted is a RAM-backed filesystem.

**Networking.** The integration between the internal and external networks of a Kubernetes cluster is another difficulty for users. We use three options to integrate the internal networks created by Kubeflow with the outside world: port-forward, load balancer and Ingress.

Kubeflow creates Istio ingress gateway service with NodePort by default, for example: 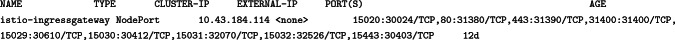


We use port-forward for quick access on a Kubernetes client. This does not require any change on the networking. 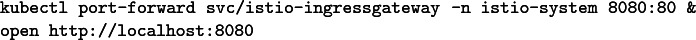


For public clouds, load balancers can be configured easily to expose Kubeflow. As Istio ingress gateway services on both ports 80 and 443, it is important to enable SSL with a signed certificate and redirect requests from port 80 to port 443 for security reasons. 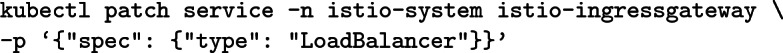


Once the service type is changed to LoadBalancer, an external IP will be assigned to the service to access Kubeflow on GCP. A host name is generated on AWS as well. 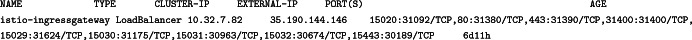


There is no load balancer configured for RKE in our private OSK cloud at present. We assign a floating IP to the Kubernetes cluster. We then configure the ingress control to access Kubeflow via the floating IP.

### Data Access

The pipelines usually have very little control over the storage for input and output. Most of Bioinformatics pipelines assume local access to POSIX-like file systems for simplicity. Kubeflow and its orchestrator Kubernetes naturally assume that pipelines use cloud-native storage as the data sources. Persistent Volumes need to be mounted as temporary storage for input and output.

We use commands (e.g., curl, wget, scp etc.) or any special clients, such as Globus or Aspera to download or upload files in the pipelines. This approach has its obvious drawbacks. This biggest issue is scalability. Data files have to be moved in batch mode and then processed. They often require large amounts of storage from Terabytes to Petabytes. As we have discussed before, the only Persistent Volume for multiple clouds is NFS. Accessing input and output data becomes moving many files into and out of NFS server for seemingly local access is very inefficient. It is impractical to move Terabytes to Petabytes of data to persistent volumes before computing.

**POSIX-like File System for Bioinformatics Applications.** We use Onedata [9] to fill the functionality gap. Onedata presents a globally federated POSIX VFS built out of local storage in Ceph, S3, NFS, Lustre, and other storage backends. There are several limitations:

1. Onedata does not support Kubernetes. There is no storage provisioner for Onedata.
2. There is only n-1 version compatibility between its client and server. Short release cycle essentially eliminates backward compatibility in practice.
3. Both client and server require root privilege.

The only viable option to bypass all the limitations above is to create Docker images with both OneClient and a Bioinformatics application. There are two options to create such merged Docker images:

1. Starting with $onedata/oneclient:{<}version\_tag{>}$ as the base image, install a Bioinformatics application. Sometimes, it is necessary to use the multi-staged build.
2. Building an image on a Docker server supporting conda, install OneClient with exactly the version as OneProvider.

To merge Samtools, we simply installed it into a given OneClient image which is fairly standard. To merge the latest version of Freebayes with OneClient, we use a two-staged build. The binaries of bamleftalign and freebayes are built from the source in a Python image first. They are then copied into OneClient image. A Dockerfile is available in the repository [10].

The second option of installing OneClient can be tricky. OneClient requires specific versions of libraries. Its installer does not do a good job to ensure that the prerequisite is met correctly.

The utility oneclient is called to mount a POSIX VFS in the container. The Bioinformatics tools will access remote files as if from a local file system as input for just-in-time data ingestion and as output for transparent write-through.

**S3-Like Cloud Storage.** S3 has firmly established its dominance as a popular cloud storage. We first tried Tensorflow API to download and upload files in S3 buckets as we were using Tensorflow/Keras in our machine learning pipeline, but switched to AWS CLI for S3 based on the Python library Boto 3 for better performance, scalability and resource consumption.

Neither AWS CLI nor Boto 3 provides official Docker images. We have created a custom image. A default Persistent Volume is used as a cache for input and output. We do not accumulate the files on the temporary storage. We download them only when they are needed, and upload them as soon as they are generated. In the pipelines, we use Kubeflow sidecar or a separate component for file transfer in parallel.

As shown in a sidecar snippet in [11], we extend the custom AWS CLI image with some simple shell scripts (image=‘davidyuyuan/aws:1000g’). We then use Kubeflow sidecar API to call our scripts for cloud storage. Both the sidecar and separate components are useful depending on whether we want to transfer files just for a single operation or shared by multiple parallel operations. They enable the classic pipelines to access cloud storage a little easier.

### Monitoring

Logging and telemetry are weak in clouds, including major public clouds. Most of the existing solutions are designed for virtual machines instead of containers (Docker), still less for container orchestration (e.g., Kubernetes) and much less for workloads (e.g., Kubeflow and pipelines on it). Our attempt to establish a cloud-agnostic solution or HPC batch processing via container orchestration adds one more challenge.

We have identified a single tool for both logging and telemetry for both VMs and Kubernetes including Kubeflow and Bioinformatics pipelines on all three clouds. Elasticsearch [12] has been known to the Open Source community for a long time. It is undeniably complex to create a traditional deployment of Elasticsearch with high availability, performance and scalability. We are using the SaaS solution and also investigating the feasibility to run Elasticsearch on Kubernetes [13]. Our goal is to let Kubernetes handle high availability, performance and scalability for a simple deployment. The details of the investigation is beyond the scope of this article. We will focus on how we make use of the SaaS solution for the pipelines on Kubeflow.

**Fig. 2.**
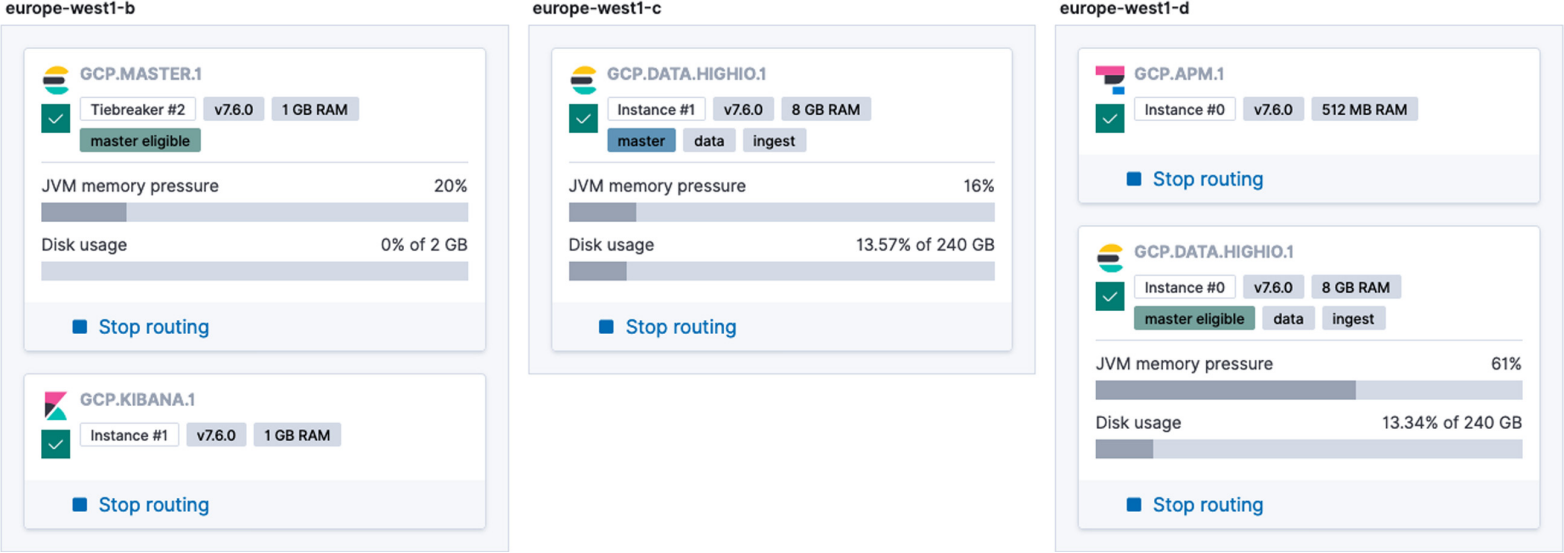
Deployment architecture of an Elasticsearch instance.

At minimum, Elasticsearch consists of Kibana, Elasticsearch, Logstash or Beats. As shown in Fig. 2, our SaaS instance contains the components on the server side: Elasticsearch and Kibana. We use Beats (Filebeat and Metricbeat) on the client side for logging and telemetry. Beats is simple to use with lower overhead compared with Logstash. DaemonSet is used to run containers (e.g., docker.elastic.co/beats/metricbeat:7.6.0) in Kubernetes clusters, instead of classic OS-specific installation packages for VMs.

**Fig. 3.**
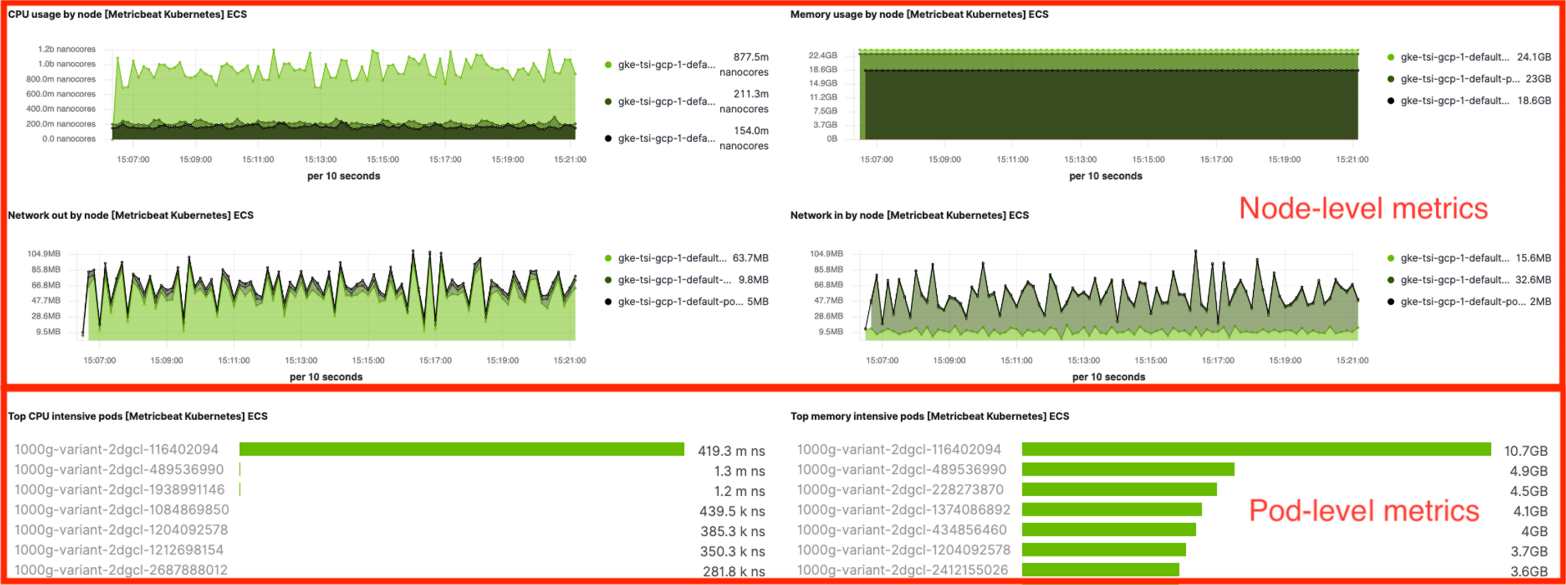
Monitoring multiple Kubernetes clusters with Elasticsearch.

This allows us to monitor clusters in different clouds in a single pane of glass, for example public GCP and private OSK in Fig. 3. Together with Filebeat container (docker.elastic.co/beats/filebeat:7.6.0), we are able to monitor both logging and telemetry of Kubernetes including Kubeflow and Bioinformatics pipelines in all three clouds.

### Using GPUs with Kubeflow

Machine Learning (ML) has wide applications in Bioinformatics, for example, genomic sequence assembly, literature analysis and image processing. Some ML pipelines take weeks to complete a training cycle, exceeding the time-limit of HPC queues. The training cycles need to be repeated many times for hyperparameter tuning.

Kubeflow runs on Kubernetes clusters with or without GPU. We position our OpenStack private cloud for pipeline development and CPU-only training. The same pipelines can be deployed as-is onto Kubeflow on GCP and AWS, where Kubernetes clusters may include GPUs. This allows us to bypass the timeout issue with HPC queues, to avoid long GPU procurement cycles, to acquire larger capacities, and to minimise the cost in public clouds.

Kubeflow includes Jupyter Notebooks by default, where they can be created with or without GPU support, depending on the initial image. In addition, Kubeflow pipeline DSL provides very handy APIs to consume GPU or TPU in a Python package:
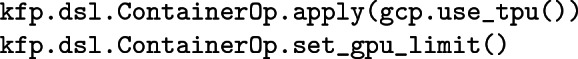


GCP and AWS provide different GPU models. They both support GPU in passthrough mode for bare metal performance. However, GCP provides separate node pools for CPUs and GPUs as well as multiple GPU pools for different GPU models in the same Kubernetes cluster. This allows us to create an ideal platform to run ML pipelines on Kubeflow.

## Result

We have successfully run two types of pipelines on Kubeflow/Kubernetes on GCP, AWS and our private OSK. Our goal is to enhance and to prove the capability of the platform for Bioinformatics. We want to make it suitable for large scale Bioinformatics research for both classic pipelines and new ML pipelines for both high throughput and high performance workloads.

1. Classic Bioinformatics pipelines - variant calling on 1000 Genomes Project [14] representing high throughput workload
2. Machine Learning pipelines - image classification on cardiomyocytes from Image Data Repository [15] representing high performance workload

### Classic Bioinformatics Pipelines

**Fig. 4.**
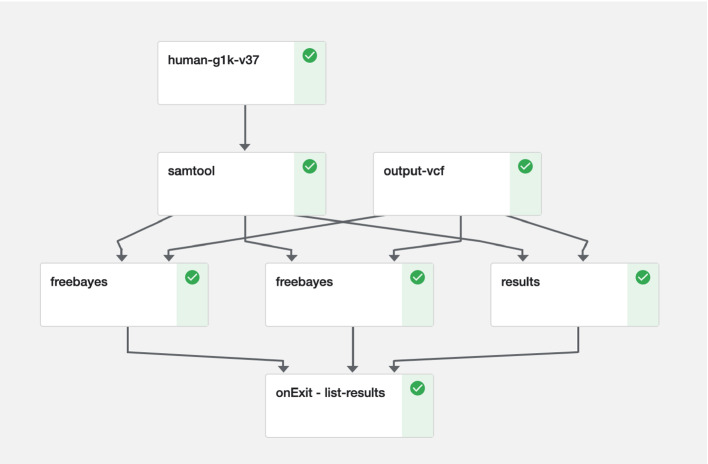
Example of a simplified classic bioinformatic workflow.

We have created a brand new pipeline, consisting of two classical tools for genomics: Samtools and Freebayes. Freebayes are to be run in parallel, one set of pods per chromosome. The output VCFs from Freebayes are cached on a shared disk, and then uploaded to an S3 bucket as soon as they arrive at the staging area (Fig. 4).

The data sources and the methods to access them are completely unchanged when we run the pipeline on GCP, AWS or the private OSK clouds:

1. Human reference genome is downloaded from an FTP server at EBI [16]. It then gets preprocessed by Samtools to generate fasta files and their indices.
2. Queries or a list of file names of the 1046 genomes is stored in an S3 bucket. It gets downloaded by a sidecar as discussed above. Freebayes is to loop through the list for each genome for each region in batches in parallel.
3. The actual alignments of the 1046 genomes are accessed with Onedata for just-in-time data ingestion from a storage volume at EBI. We have discussed details on how to integrate Onedata with Kubernetes above.

**Fig. 5.**
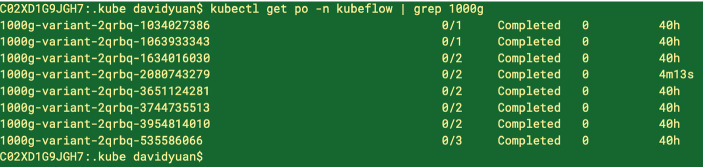
Pods for the pipeline scale up and down efficiently as needed.

A complete run of 1046 genomes on all 26 regions takes several weeks. We usually scale down to three fastest regions (‘GL000207.1’, ‘MT’, ‘Y’) for a 40-hour-run (Fig. 5). The exit handler gets invoked by Kubeflow where we have only implemented a simple logic to list all the VCFs uploaded to the S3 bucket (onExit - list-results).

One point worth noting is that Kubeflow uses Python as the programming language for pipelines. It provides developers much needed lexicon to construct DAG with simple expressions and function calls in an extremely condensed and elegant style [17].

### Machine Learning Pipelines

Kubeflow is designed to provide the first class support for Machine Learning. As shown in the diagram in Kubeflow overview [1], tools and services needed for ML have been integrated into the platform, where it is running on Kubernetes clusters on public and private clouds.

A set of the most popular ML tools, such as Jupyter, TensorFlow, PyTorch, MPI, XGBoost, MXNet, etc., are included. We used Jupyter and TensorFlow for our ML pipeline. The Kubeflow applications and scaffolding integrates the ML tools with the underlying Kubernetes cluster supported by various clouds, in our case: GCP, AWS and OpenStack on premises. There are also other components providing service mesh, programming model, instrumentation, influencing, etc. to make the platform fully operational for both experimental and production phases.

We have created a notebook for image classification. The images are whole slides of cardiomyocytes published in 2018 [18]. The public data is stored in the IDR hosted by EBI. We have decided to use our Kubeflow on GCP with GPU support to speed up the model training for high performance. A notebook server is created with an Docker image with Tensorflow 2.1.0 and GPU support accordingly.

**Fig. 6.**
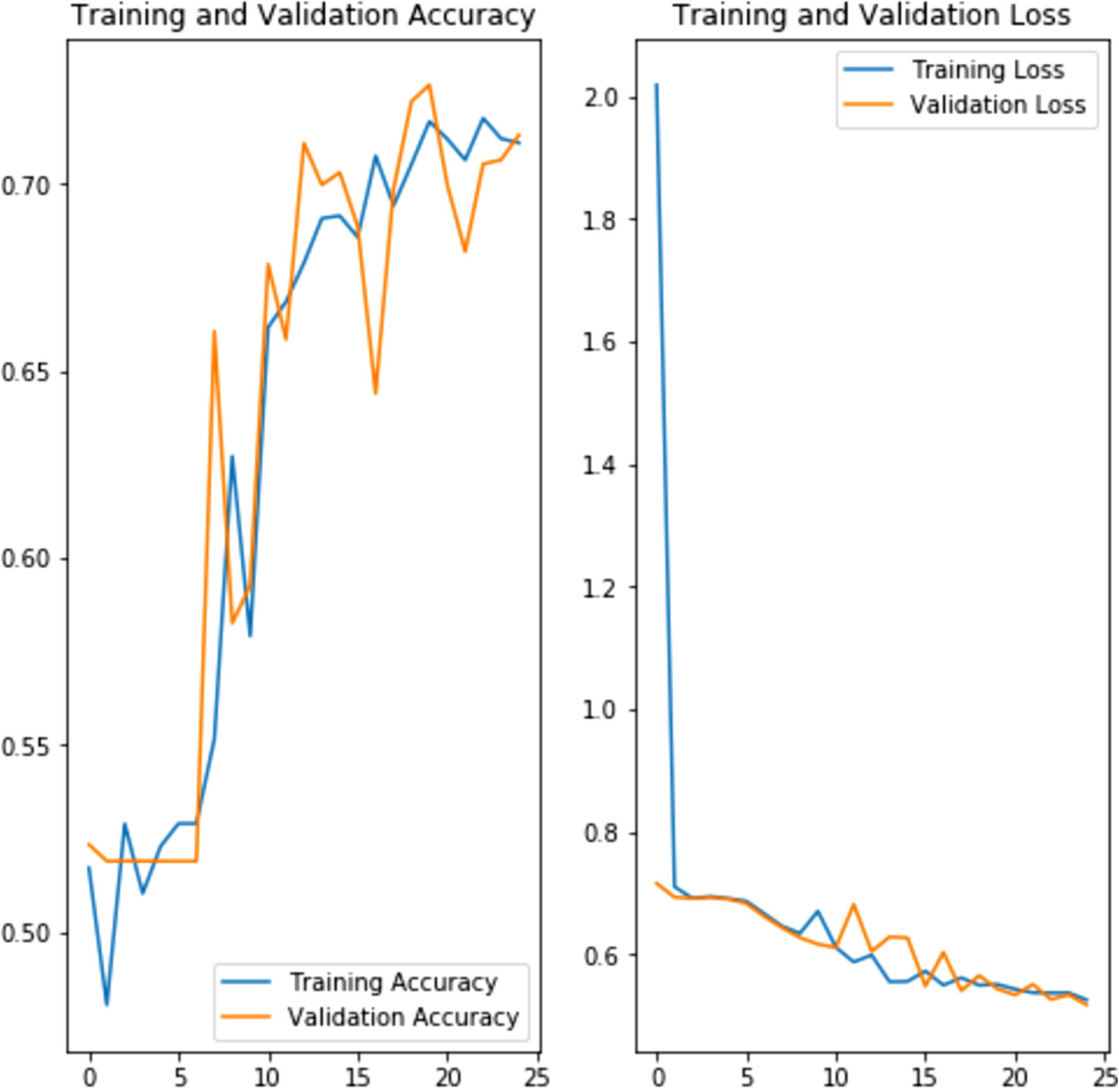
Training and validation of a CNN model with cardiomyocytes images on Kubeflow

We use the latest OMERO 5.6.0 JSON API [19] to download the images. There is a limit on the IDR server of maximum downloads of 1000 images, which gives us 1978 images to work with, comparable to the original datasets of 2277 usable images. This is on the smaller side for CNN training and validation. The image quality and annotation are good so it gives us satisfying results (Fig. 6).

The training and validation with GPU are surprisingly fast with 4s for each epoch on the original images and 8 s for augmented images. With the per second billing on both GCP and AWS together with dynamic resource allocation on Kubeflow, the cost to run ML pipelines with GPUs is very low. This fully cloud-native and cloud-agnostic approach provides advantages not only over HPC on premises but also over HPC-in-the-cloud, where GPUs still have to be reserved for the lifecycle of the job, whether they are used well or not.

## Conclusion

We have successfully run pipelines on Kubernetes in OpenStack, Google Cloud Platform and Amazon Web Services, in particular, on Kubeflow with more sophisticated job scheduling, workflow management, and first class support to machine learning. We choose Kubeflow/Kubernetes to avoid the overhead of provisioning of virtual machines, to achieve rapid scaling with containers, and to be truly cloud-agnostic in all three cloud environments.

We have chosen two very typical pipelines in Bioinformatics: one for genomic sequence analysis and the other for image classification; one for classic tools and the other for modern machine learning; one for high throughput and the other for high performance; one for classic pipeline and the other for Jupyter notebook. With the successful deployment of these two pipelines, we can conclude confidently that Kubeflow can satisfy complex requirements by Bioinformatics.

Kubeflow and Kubernetes have also introduced interesting challenges. We have systematically analysed and addressed various aspects in deployment, storage and networking. We have identified and implemented methods to access data for input and output in CLI and Python APIs. We have successfully proposed and implemented a creative solution to combine the strength of Onedata and Docker for the just-in-time data ingestion as well as transparent write-through. For S3 storage, we have created a custom AWS CLI image and run the container as either a sidecar or a separate operation for parallel operations to transfer objects. We also have integrated Elasticsearch for both logging and telemetry. By adding GPU to the Kubernetes cluster, and then to Jupyter notebook server, we are able to train a CNN model in seconds per epoch.

With the excellence in Kubeflow and Kubernetes frameworks and our solutions to compensate for their limitations, we are able to run both high throughput and high performance pipelines at scale. We are confident that it is feasible to run Bioinformatics pipelines efficiently via container orchestration in all major clouds with excellent portability. Jobs in different pipelines or between different runs are now able to share cloud resources efficiently, much better than traditional HPC-in-the-cloud solutions.
